# Erratum to: *BMC Palliative Care*, Vol. 17

**DOI:** 10.1186/s12904-017-0233-9

**Published:** 2017-10-10

**Authors:** Pilot and Feasibility Studies

**Affiliations:** BMC Palliative Care, London, UK

An error occurred during the publication of a number of articles in *BMC Palliative Care*. These articles were erroneously published in volume number 17, which is listed with a publication date of 2018. However, these articles were published in final form in the year 2017.

Provided below are the actual publication dates and the correct citation for each affected article. Please note that the citation refers to volume number 17 with the year as 2018.

Article: 10.1186/s12904-017-0214-z

Publication date: 14 July 2017.

Correct citation: Firn et al. BMC Palliative Care (2018) 17:7. 10.1186/s12904-017-0214-z

Article: 10.1186/s12904-017-0221-0

Publication date: 14 July 2017.

Correct citation: Fox et al. BMC Palliative Care (2018) 17:9. 10.1186/s12904-017-0221-0

Article: 10.1186/s12904-017-0215-y

Publication date: 13 July 2017.

Correct citation: Wakeham et al. BMC Palliative Care (2018) 17:8. 10.1186/s12904-017-0215-y

Article: 10.1186/s12904-017-0220-1

Publication date: 11 July 2017.

Correct citation: Veigh et al. BMC Palliative Care (2018) 17:6. 10.1186/s12904-017-0220-1

Article: 10.1186/s12904-017-0218-8

Publication date: 10 July 2017.

Correct citation: Wang et al. BMC Palliative Care (2018) 17:5. 10.1186/s12904-017-0218-8

Article: 10.1186/s12904-017-0217-9

Publication date: 10 July 2017.

Correct citation: Llobera et al. BMC Palliative Care (2018) 17:4. 10.1186/s12904-017-0217-9

Article: 10.1186/s12904-017-0219-7

Publication date: 6 July 2017.

Correct citation: Orellana-Rios et al. BMC Palliative Care (2018) 17:3. 10.1186/s12904-017-0219-7

Article: 10.1186/s12904-017-0210-3

Publication date: 3 July 2017.

Correct citation: Pesut et al. BMC Palliative Care (2018) 17:2. 10.1186/s12904-017-0210-3

Article: 10.1186/s12904-017-0213-0

Publication date: 21 June 2017.

Correct citation: Reeve et al. BMC Palliative Care (2018) 17:1. 10.1186/s12904-017-0213-0

Since this error was detected, the journal has resumed publication in the 2017 volume, volume number 16. In 2018, the journal will resume publication in volume number 17. The publisher apologizes for any confusion caused by this error.

